# Assessment of Bacterial Inoculant Delivery Methods for Cereal Crops

**DOI:** 10.3389/fmicb.2022.791110

**Published:** 2022-01-26

**Authors:** Yen Ning Chai, Stephanie Futrell, Daniel P. Schachtman

**Affiliations:** Department of Agronomy and Horticulture and Center for Plant Science Innovation, University of Nebraska – Lincoln, Lincoln, NE, United States

**Keywords:** inoculation, plant growth promoting bacteria, rhizosphere, endosphere, *Chitinophaga*, *Caulobacter*, *Terrabacter*, sorghum

## Abstract

Despite growing evidence that plant growth-promoting bacteria can be used to improve crop vigor, a comparison of the different methods of delivery to determine which is optimal has not been published. An optimal inoculation method ensures that the inoculant colonizes the host plant so that its potential for plant growth-promotion is fully evaluated. The objective of this study was to compare the efficacy of three seed coating methods, seedling priming, and soil drench for delivering three bacterial inoculants to the sorghum rhizosphere and root endosphere. The methods were compared across multiple time points under axenic conditions and colonization efficiency was determined by quantitative polymerase chain reaction (qPCR). Two seed coating methods were also assessed in the field to test the reproducibility of the greenhouse results under non-sterile conditions. In the greenhouse seed coating methods were more successful in delivering the Gram-positive inoculant (*Terrabacter* sp.) while better colonization from the Gram-negative bacteria (*Chitinophaga pinensis* and *Caulobacter rhizosphaerae*) was observed with seedling priming and soil drench. This suggested that Gram-positive bacteria may be more suitable for the seed coating methods possibly because of their thick peptidoglycan cell wall. We also demonstrated that prolonged seed coating for 12 h could effectively enhance the colonization of *C. pinensis*, an endophytic bacterium, but not the rhizosphere colonizing *C. rhizosphaerae*. In the field only a small amount of inoculant was detected in the rhizosphere. This comparison demonstrates the importance of using the appropriate inoculation method for testing different types of bacteria for their plant growth-promotion potential.

## Introduction

Plants and soil microbiomes have interacted and co-evolved for over a million years. Many soil-inhabiting microbes are capable of improving plant growth (Delaux and Schornack, 2021). For example, arbuscular mycorrhizal fungi and certain bacteria improve plant nutrient uptake (Parniske, 2008; Zaidi et al., 2009; Santi et al., 2013; Lopes et al., 2021b), biocontrol microbes suppress plant pathogens (Weller, 2007), while certain bacteria produce phytohormones to improve plant growth (Egamberdieva et al., 2017). To facilitate close interactions with these microbes, plants release rhizodeposits from plant roots into the rhizosphere, a soil layer adhering to the root, to serve as carbon sources and also as signaling cues to these microbes (Mendes et al., 2013). Apart from interacting with plants, some microbes can further colonize the inner root zone termed endosphere and those microbes are known as root endophytes. The intimate association between root endophytes and root tissues may enhance the exchange of nutrients between plants and microbes (Harman and Uphoff, 2019). For instance, the colonization of rhizobia inside root nodules allows these bacteria to fix N more efficiently due to the hypoxic conditions in the nodules, the fixed N is then supplied to the host plant in exchange for carbon (Ledermann et al., 2021).

Due to the advantages conferred by plant growth-promoting bacteria on plant vigor, using these bacteria as bioinoculants can potentially substitute or supplement chemical fertilizers that bring many adverse effects on the environment (Santos et al., 2019). The method of inoculation is an important factor that can affect the colonization of the inoculant in the host plant and impact its downstream effect on plant growth (Ciccillo et al., 2002; Müller and Berg, 2007; Fukami et al., 2016; O’Callaghan, 2016; Vassilev et al., 2020; Lopes et al., 2021b). Numerous methods have been used to deliver microbes to host plants, including soil drench, seed inoculation, and plant inoculation (Rocha et al., 2019). Seed inoculation is the most widely used on a commercial scale since it is suited to agricultural production and requires less inoculant than the other two under field conditions. To enhance the survival of the bacteria coated on the seeds, a carrier such as peat slurry or a film coat consisting of alginate polymers are often mixed with bacteria during the coating process as a layer protecting inoculants from environmental stresses such as desiccation and temperature perturbations (O’Callaghan, 2016; Lobo et al., 2019; Santos et al., 2019). Soil drench or in-furrow inoculation, on the other hand, is performed by applying the inoculants in soil before or after planting (Campo et al., 2010; Hungria et al., 2013). It has several advantages over seed inoculation as it prevents the inoculants from being inhibited by the chemicals coated on seeds (e.g., fungicides and pesticides) and can be used to deliver inoculants at higher density without being constrained by seed size (Rocha et al., 2019). However, this method is relatively impractical for field-scale compared to seed coating because higher inoculant concentration is usually required for soil inoculation to obtain desirable outcomes for plant growth (Rocha et al., 2019). Foliar spray and root dipping are two of the most commonly used methods for plant inoculation (Rocha et al., 2019). Plant inoculation is usually performed at the seedling stage because the earlier the inoculant colonizes the plant, the more likely it can persist in the plant tissues even if the plant is later colonized by other microbes (Carlstrom et al., 2019; Wippel et al., 2021). One of the greatest advantages of seedling inoculation is that it greatly enhances the plant colonization of the inoculant, but it also has the drawback of being highly impractical for use under field conditions.

Commercialization of bioinoculants began in the late 1980s and microbial inoculants have been widely applied in India and South America, especially Brazil where approximately 78% of crops planted are inoculated annually (Santos et al., 2019). Among these commercial bioinoculants, *Pseudomonas* and *Bacillus* are the most commonly used while rhizobia are the most studied bacterial inoculants (Rocha et al., 2019). Rhizobia are not only commonly used to improve the productivity of leguminous plants as they can establish endosymbiotic relationships with legumes to fix nitrogen (Andrews and Andrews, 2017), but also have the potential to enhance non-legume growth since many of them possess other plant growth-promoting abilities such as phosphorus solubilization and phytohormone production (García-Fraile et al., 2012). Despite being widely studied, rhizobial inoculants suffer from the drawback of having a short shelf life especially when coated on seeds (O’Callaghan, 2016). Endospore-forming bacteria like *Bacillus* are often preferred as seed inoculants as they can better withstand unfavorable conditions (Price et al., 2010). Gram-positive bacteria which have thicker peptidoglycan layer on their cell wall are also good for bioinoculants because the cell wall renders them less susceptible to desiccation in the seed coating process compared to the Gram-negative bacteria (Viaene et al., 2016; Xu et al., 2018). *Pseudomonas* strains, despite being non-spore-forming and Gram-negative, are often used against phytopathogens such as *Pythium* and *Fusarium* due to their biocontrol properties (O’Callaghan, 2016). Although there are many bioinoculants with different plant growth-promoting potentials, the methods for delivering these bacteria under greenhouse conditions for basic research have not been compared or published.

Three bacteria isolated from field-grown sorghum were used in this study (Chai et al., 2021), with *Chitinophaga pinensis* (Gram-negative) originating from the root endosphere while *Caulobacter rhizosphaerae* (Gram-negative) and *Terrabacter* sp. (Gram-positive) were from the soil. Despite being widely distributed and abundant in the soil and/or rhizosphere of various crops, the genera *Chitinophaga* (Chung et al., 2012; Li et al., 2014; Chiniquy et al., 2021) and *Caulobacter* (Gao et al., 2018; Lopes et al., 2021a) are rarely tested for their plant growth-promoting abilities. Compared to these two genera, the genus *Terrabacter* has also been detected in many plant species, including maize (Dohrmann et al., 2013), sorghum (Lopes et al., 2021a), and napa cabbage (Bhattacharyya et al., 2018), but in very low abundance and is understudied. Therefore, we sought to determine whether these bacteria could promote plant growth and their host colonization efficiency with different inoculation methods since they are phylogenetically distinct and exhibit different cell wall structure.

In this study, we used *Sorghum bicolor* which is the fifth most widely grown cereal crop in the world to compare five bacterial inoculation methods. Sorghum is widely grown in marginal environments where microbial inoculation may provide strong benefits, particularly on parts of the African continent where inputs such as fertilizer are scarce (Tonitto and Ricker-Gilbert, 2016). Our aim was to compare seedling priming, soil drench, and three seed coating methods (direct seed coating, alginate seed coating, and 12-h coating) for their efficacy of delivering three different bacterial strains to sorghum under sterile and field conditions. While it is possible to find these methods in the literature (Lopes et al., 2021b), a direct comparison under the same conditions along with a molecular analysis is not available. Our findings highlight the importance of tailoring the inoculation method to the specific type of bacteria being studied to get optimal plant growth-promoting results.

## Materials and Methods

### Bacteria Strains

*Chitinophaga pinensis* isolated from sorghum root endosphere, as well as *Terrabacter* sp. and *C. rhizosphaerae* isolated from soil where sorghum was growing, were used for inoculation in this study. These bacteria have been used in a previous study (Chai et al., 2021). The draft genome sequences and gene annotations of these bacteria are available through the IMG portal at the Joint Genome Institute under the taxon ID 2818991442, 2818991454, and 2818991462, for *C. pinensis*, *C. rhizosphaerae*, and *Terrabacter* sp., respectively.

### Sorghum Seed and Potting Mix Sterilization

A sweet sorghum variety, Grassl, was used throughout this experiment (Boyles et al., 2019). Grassl seeds were surface-sterilized for 6 h with chlorine gas generated by adding 3.3 ml of hydrochloric acid to 100 ml of sodium hypochlorite in a desiccator. Surface-sterilized seeds were then washed with sterile water and plated on YPD medium (Costanzo et al., 2001) to verify that there were no bacteria on the seed surface. The potting mix used in the greenhouse experiment consisted of two parts of peat and one part of vermiculite. To sterilize the pot and potting mix, 325 g of the potting mix were added to a pot with a diameter of 12.7 cm and autoclaved three times. After autoclaving, the potting mix was plated on YPD to ensure there were no viable microbes.

### Bacteria Inoculation

All bacteria were grown in R2A broth (Reasoner and Geldreich, 1985) except for *C. rhizosphaerae*, which was grown in peptone-yeast extract broth (Hottes et al., 2004) because it did not grow well in R2A. Two days before planting, each bacterial strain was grown on a rotary shaker at 180 rpm at room temperature (24°C). After a day of growth, a portion of each liquid culture was transferred to a fresh medium to allow for continued growth. On the day of the experiment, each bacterial culture was pelleted at 4,000 rpm for 10 min and resuspended in phosphate-buffered saline (PBS, 8 g/L NaCl, 0.2 g/L KCl, 1.44 g/L Na_2_HPO_4_, and 0.24 g/L KH_2_PO_4_). The optical density (OD) of each of the bacterial/PBS suspensions was measured at 600 nm with a spectrophotometer and adjusted to an OD_600_ of 1 that corresponded to 10^9^ colony forming units (CFUs) for each of these bacteria before inoculation. The CFU number was derived by plating 200 μl of diluted bacterial cultures with an OD_600_ of 1 on R2A medium.

#### Soil Drench

Soil drench was performed with the bacterial suspension 1 day after the plant shoot emerged from the soil. One part of each bacterial solution was added to 69 parts of 1 × plant nutrient solution (Hoagland and Arnon, 1950) to achieve a final OD_600_ of 0.002. Bacteria/nutrient mix equivalent to 30% of the soil volume was then added to each pot in a laminar flow hood.

#### Direct Seed Coating

Surfaced-sterilized seeds were dipped into the bacteria suspension in PBS and air-dried for 20 min in the laminar flow hood before planting.

#### Twelve Hours Seed Coating

Surface-sterilized seeds were immersed in bacteria suspension in PBS and put on a rotary shaker shaking at 180 rpm for 12 h at room temperature and air-dried for 20 min in the laminar flow hood before planting.

#### Alginate Seed Coating

Surface-sterilized seeds were dipped into bacteria suspension in 2% (wt/vol) alginate followed by transferring the seeds into 0.1 M CaCl_2_ to solidify. The alginate-coated seeds were then air-dried in the laminar flow hood for 20 min before planting.

#### Seedling Priming

Surface-sterilized seeds were germinated at 30°C in a sterilized petri dish with wet filter paper for 24 hours. When seeds germinated they were carefully transferred to a new petri dish filled with bacteria suspension in PBS and placed on a rotary shaker at 20 rpm for 12 h. The inoculated seedlings were then sowed carefully in soil in a laminar flow hood.

#### No Microbe Control

In a laminar flow hood, 1:69 of PBS in 1X Hoagland solution was added to each pot right after germination.

To measure the concentration of viable bacteria on inoculated sorghum seeds, 10 inoculated seeds were placed in 10 ml of PBS and vortex vigorously for 10 min followed by a 4-fold serial dilution in PBS. About 200 μl of each dilution was then plated on R2A medium and allowed to grow at room temperature. Approximately 10^3^–10^4^ CFU per seed were detected for the three bacteria with seed inoculation.

### Experimental Design

#### Greenhouse Experiment

This experiment was comprised of a total of 240 pots (three bacterial strains × five inoculation methods × five replicate pots × three sampling time points + five uninoculated control × three sampling time points). Pots were planted on March 1, 2019. In a sterile laminar flow hood, three seeds were planted into the sterile soil in each pot and the pots were covered with saucers before transferring to the greenhouse to minimize airborne contamination. Pots were arranged in the greenhouse in a completely randomized design. Seedlings were thinned to one plant per pot and a small hole was made on each saucer covering the pot to allow for shoot growth. The greenhouse was 27°C during the day and 21°C at night, with a photoperiod of 16 h. Sterilized water and 1X Hoagland nutrient solution were applied to each pot to keep the soil evenly wet through a sterile plastic tube into the hole on the saucers covering the pot. Three samplings were conducted at 4-, 6-, and 8-week after planting. For each harvest, fresh and dry weights of both shoot and root were measured. Roots were washed to remove the soil prior to weighing. To obtain the dry weight, fresh plant material was dried in an oven at 60°C for 3 days. Rhizosphere and root tissues were collected for qPCR analysis to quantify the colonization of inoculated microbes.

#### Field

Grassl seeds were inoculated with each of the three bacteria using alginate and 12 h coating and were planted in a field (40.85475, −96.61) on June 1, 2019. The field soil was a silty loam with 3.9% organic matter, and the concentrations of some major chemical components of the soil were: pH: 5.55; 28 ppm nitrate-N; 446 ppm potassium; 10.7 ppm sulfate; 1,657 ppm calcium; and 253 ppm magnesium. Seed inoculation was carried out on the day before planting and planted immediately the next morning. A total of 64 plots (3.7 m × 1.5 m) were included in this experiment (three bacteria and no-microbe control × two inoculation methods × eight replicates) and arranged in complete randomized design. Early in the field study, 78.5 kg/ha of nitrogen fertilizer was applied in the form of urea. Weeding was carried out frequently and a weed score was assigned for each plot early on during the experiment with a range from 0 to 3, with the score of zero indicating no weeds and three designating severe weed infestation. The rhizosphere and root samples were collected twice, once at the vegetative (July 10, 2019) stage and a second time at flowering (August 23, 2019) for qPCR analysis to quantify the number of bacteria colonizing the rhizosphere and root. Shoot fresh weight and dry weight were measured twice during the course of the experiment. Biomass was measured on July 10 (30 days after germination) and October 8, 2019 (120 days after germination). Additionally, grain yield was also measured in October.

### Sampling and Processing of Rhizosphere and Root Tissue for qPCR

#### Sampling

To collect root and rhizosphere, we removed the bulk soil from the root system, chose a range of root types, and put them in a 50 ml tube filled with 35 ml of phosphate buffer (6.33 g/L NaH_2_PO_4_ and 8.5 g/L Na_2_HPO_4_ anhydrous) supplemented with 0.01% of Silwet and shook them vigorously for 3 min on a vortexer. The roots were then transferred to a clean 50 ml tube, the remaining phosphate buffer with rhizosphere soil was collected (McPherson et al., 2017).

#### Rhizosphere Processing

The rhizosphere soil samples were filtered through a sterile 100 μm mesh filter unit (Fisher Scientific, United States) into a clean 50 ml tube and pelleted at 6,000 × *g* for 5 min at room temperature using a centrifuge. The pellet was resuspended in 1.5 ml phosphate buffer and transferred to a sterile 2 ml tube. The rhizosphere was re-pelleted by spinning tubes for 2 min at full speed. The supernatant was drained from the tube and stored at −20°C until DNA extraction.

#### Root Processing

Roots were surface sterilized by rinsing for 1 min in 50% sodium hypochlorite + 0.005% Tween 20, followed by a 1 min rinse in 70% ethanol, and three rinses in sterile ultrapure water for 1 min each. Roots were blotted dry, placed in a 2 ml microfuge tube, and frozen at −80°C prior to being ground in liquid N for DNA extraction.

### DNA Extraction of the Rhizosphere, and Root Samples

Rhizosphere DNA was extracted using MagAttract® PowerSoil®DNA KF Kit (Qiagen) and root DNA using MagMAX™ Plant DNA Kit (ThermoFisher Scientific), with a KingFisher Flex Robot (ThermoFisher Scientific) following the manufacturer’s protocol. DNA concentration was quantified using QuantiFluor® dsDNA System (Promega) with CLARIOstar® Plus microplate reader (BMG LABTECH) following the manufacturer’s protocol.

### qPCR

Different primer pairs were used for each bacterium to provide adequate specificity for each of the three bacteria used in this study. These primer pairs were constructed from the corresponding genome sequence of each isolate (Table 1). Standard curves were constructed by serial dilutions of the genomic DNA of each bacterium from 10^6^ to 10^1^ pg DNA μl^−1^ using molecular grade water. The genome copy number of each bacteria was computed using their genome sizes, (*C. rhizosphaerae*: 5563326 bp, *Terrabactor* sp.: 4320267 bp, and *C*. *pinensis*: 8318214 bp), DNA molecular weight of 650 Da bp^−1^, and Avogadro’s constant of 6.022 × 10^23^. The detection limit of *C. rhizosphaerae*, *C*. *pinensis*, and *Terrabacter* sp. were 13, 7, and 7 genome copies, respectively. All qPCR was carried out using CFX Connect (Bio-Rad Laboratories Inc., Hercules, CA, United States) in a final volume of 10 ml, which contained 5 ml of Power Sybr Green PCR Master Mix (Applied Biosystems, Foster City, CA, United States), 0.5 ml of each of the forward and reverse primers (10 pM each), 1 ng of template DNA, and water. The same amplification conditions were used for all three bacteria with an initial incubation at 95°C for 10 min, followed by 40 cycles of denaturation at 95°C for 15 s, annealing at 60°C for 30 s, and extension at 72°C for 15 s. The specificity of amplification was determined using a melting curve analysis at the end of the amplification by ramping the temperature up to 95°C for 1 min followed by a 0.5°C s^−1^ increment from 60 to 95°C. Three technical replicates were performed for each sample in the qPCR. The average of the three Ct values from the technical replicates was calculated and reported.

**Table 1:** Primer pairs used to amplify the three bacterial isolates.

	Primer sequence (5'–3')	Amplicon size (bp)
*Chitinophaga pinensis*		
1204_1F	TTCCGTGCCTCATACTCAGA	157
1204_1R	CCTCAGGAGCAAGTCCATTC	
*Caulobacter rhizosphaerae*		
3260_2F	GCTTCAACTTAGGCCTGTCG	150
3260_2R	GGGCGGTCTACTAAACATCG	
*Terrabacter* sp.		
3264_2F	ATTCAAGTGCATGGTGAACG	165
3260_2R	GTCAAAGCCACAGTCGATGA	

### Statistical Analysis

All the statistical analyses in this study were performed using R v3.6.0 (R Developmental Core Team, 2018). A one-way ANOVA was performed to determine whether the colonization of the inoculated bacteria (logarithm of bacterial copy number) in the rhizosphere and root endosphere was influenced by inoculation method. Tukey’s HSD *post hoc* pairwise comparison was then conducted to compare the mean difference between inoculation methods. These analyses were performed on both the greenhouse and field datasets.

Linear models were constructed using *lm* function to determine the changes in sorghum shoot and root dry biomass for each combination of inoculation method, bacterial strain, sampling time point, and degree of colonization (log copy number). Prior to model construction, root and shoot dry weight were power-transformed by 0.222 and 0.303, respectively, which were determined using *boxcox* function in “MASS” package (Venables and Ripley, 2002) to homogenize the residual variances. Backward selection was performed to eliminate the interactions that were not significant in affecting sorghum biomass from the global models. The marginal means for each treatment and strain combination were computed and subjected to Tukey’s HSD pairwise comparisons using the *emmeans* function in “emmeans” package (Lenth, 2021). Plots were generated using *ggboxplot* and *ggplot* function in “ggpubr” (Kassambara, 2020) and “ggplot2” package (Wickham, 2016), respectively.

## Results

### Primer Specificity

To construct primer pairs specific for each bacterial isolate, we first mapped each genome sequence to the NCBI database to identify the genomic regions that were unique to each of the three bacterial isolates and not found in their close relatives. As a result, we identified the genomic regions that exhibited zero matches when searched using the BLAST alignment tool. Using these unique genomic regions, we constructed three primer pairs for these isolates (Table 1). We further confirmed the specificity of these primers on the targeted strains by performing specificity tests on their closely related isolates in our culture collection from sorghum in the same field, some of which have a perfect match (100% similarity) with our targeted bacteria in their full-length 16S rRNA regions (Table 2; Supplementary Table 1).

**Table 2:** Bacterial strains used to test the specificity of each primer pair.

Bacteria strain	Similarity of 16S rRNA to the targeted strain	Primer tested	Amplification	Origin
*Caulobacter segnis* 1776	97%	3260_2F, 2R	Not detected	Isolated from sorghum rhizosphere
*Caulobacter rhizosphaerae* 2154	100%		Not detected	Isolated from sorghum soil
*Chitinophaga pinensis* 1232	100%	1204_1F, 1R	Not detected	Isolated from sorghum root from low-nitrogen field
*Chitinophaga sancti* 3198	97%		Not detected	Isolated from sorghum root from low-nitrogen field
*Chitinophaga pinensis* 1209	100%		Not detected	Isolated from sorghum root from low-nitrogen field
*Terrabacter* sp. 3211	99%	3264_2F, 2R	Not detected	Isolated from sorghum root from full-nitrogen field
*Terrabacter lapilli* 3265	98%		Not detected	Isolated from sorghum root from full-nitrogen field

### Quantification of Bacterial Colonization in Rhizosphere and Root Endosphere Under Sterile Greenhouse Conditions

**Figure 1:**
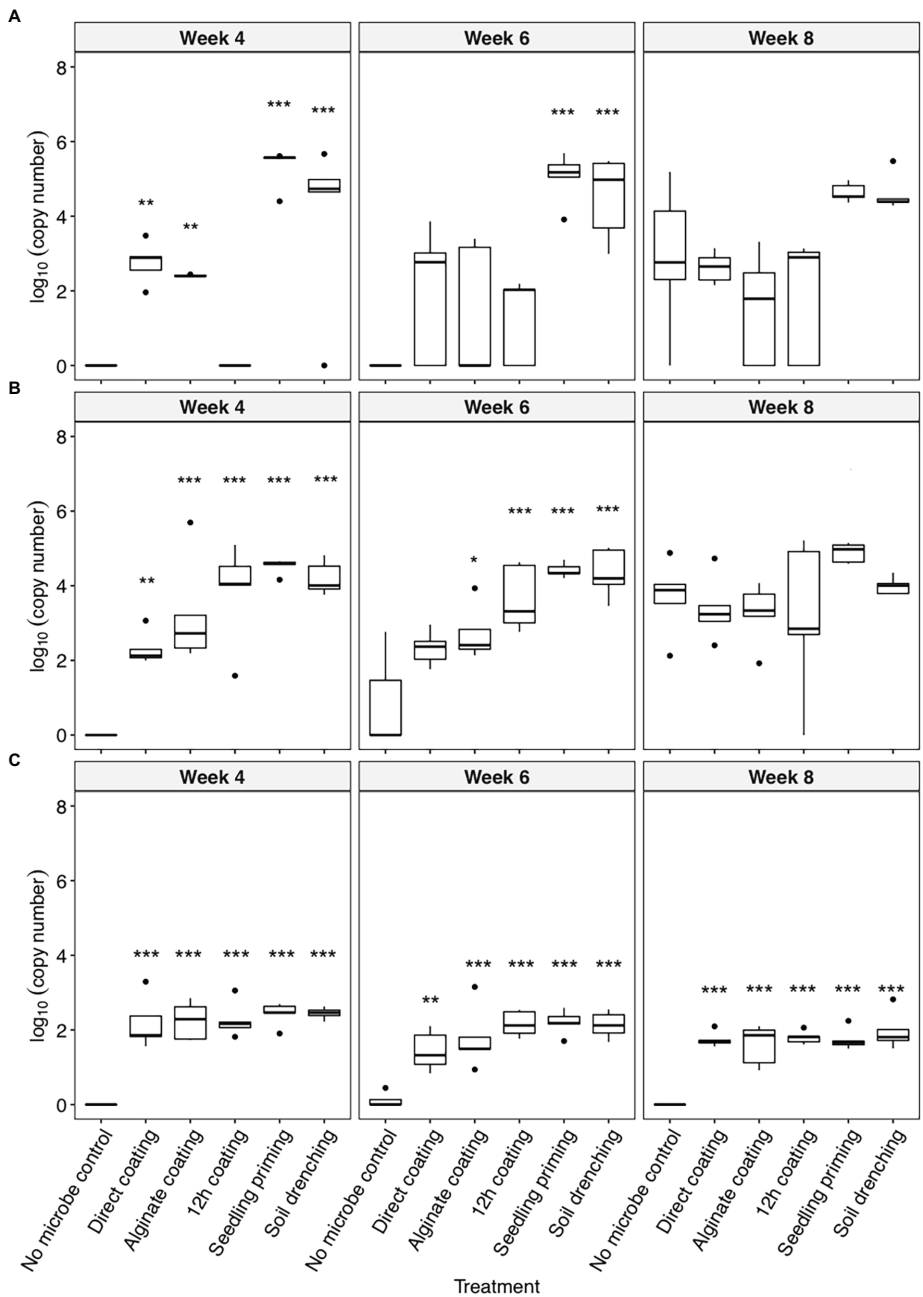
The colonization [log_10_(bacterial DNA copy number)/ng of rhizosphere DNA] of **(A)** *Caulobacter rhizosphaerae*, **(B)** *Chitinophaga pinensis*, and **(C)** *Terrabacter* sp. in the rhizosphere of *Sorghum bicolor* inoculated using different methods at week 4, 6, and 8 after planting. ANOVA was performed with Tukey’s HSD correction for multiple comparisons. Asterisks denote significant difference in the bacteria DNA copy number between inoculated samples and uninoculated controls. ^*^p ≤ 0.05, ^**^p ≤ 0.01, ^***^p ≤ 0.001.

All three bacteria were detectable in the rhizosphere of the inoculated plants up to 8 weeks after planting (Figure 1). The colonization of *C. rhizosphaerae* in the rhizosphere was greater (10^4^–10^5^ copies per ng of rhizosphere DNA) when the seedling priming and soil drench method were used as compared to the seed coating approaches (10^2^–10^3^ copies per ng of rhizosphere DNA; Figure 1A). Similar trend was also found for *C. pinensis* where seedling priming and soil drench method promoted its colonization in the rhizosphere (Figure 1B). Prolonged seed coating for 12 h enhanced the colonization of *C. pinensis* (Figure 1B) but did not improve the colonization of *C. rhizosphaerae* in the rhizosphere (Figure 1A). The colonization of *Terrabacter* sp. in sorghum rhizosphere was consistent in all five inoculation methods; although its abundance was lower (10^2^ copies per ng of rhizosphere DNA) as compared to the other two strains which reached as high as 10^5^ copies per ng of rhizosphere DNA (Figure 1C). *C. pinensis* and *C. rhizosphaerae* but not *Terrabacter* sp. were detected in the rhizosphere of the uninoculated control at week 8 after planting (Figures 1A,B).

**Figure 2:**
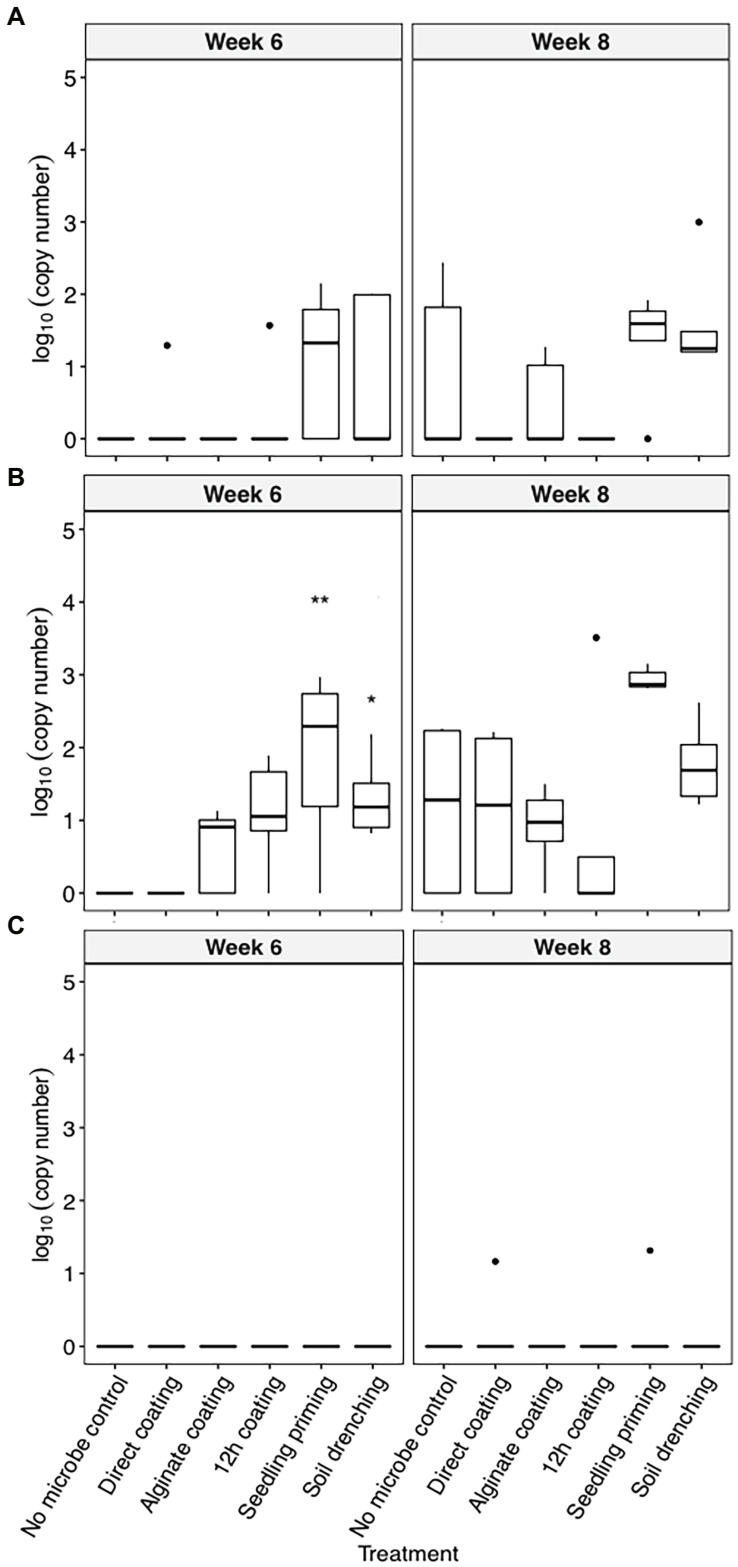
The colonization [log_10_(bacterial DNA copy number)/ng of root endosphere DNA] of **(A)** *C. rhizosphaerae*, **(B)** *C. pinensis*, and **(C)** *Terrabacter* sp. in the root endosphere of *S. bicolor* inoculated using different methods at week 4, 6, and 8 after planting. ANOVA was performed with Tukey’s HSD correction for multiple comparisons. Asterisks denote significant difference in the bacteria DNA copy number between inoculated samples and uninoculated controls. ^*^p ≤ 0.05, ^**^p ≤ 0.01.

*C. pinensis* was the only strain that could robustly colonize the root endosphere starting from week 6 after planting (Figures 2A–C). The colonization of *C. pinensis* in the root endosphere was greater when inoculated with seedling priming and soil drench as compared to the seed coating methods at week 6 after planting (Figure 2B). *C. pinensis* was detected in the root endosphere of the uninoculated plants at week 8.

### The Effect of Bacterial Isolate Inoculation on Plant Growth Under Sterile Greenhouse Condition

**Figure 3:**
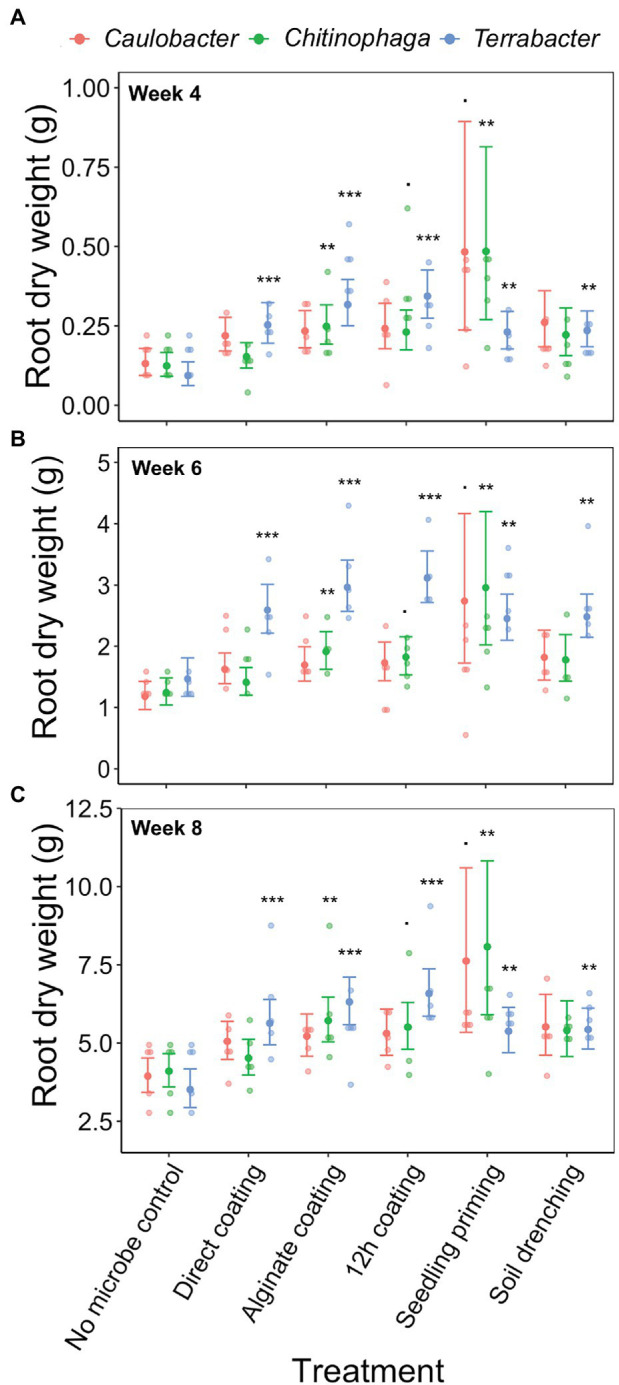
Effects of bacterial inoculation using different inoculation methods on *S. bicolor* root dry weight at week **(A)** 4, **(B)** 6, and **(C)** 8 after planting. Root dry weight was fitted to linear model and Tukey’s HSD correction was performed for multiple comparisons. Error bars and center points denote the 95% CIs and the marginal means for each strain and inoculation method combination, respectively, derived from the linear model. The distribution of raw data is represented by the dots. ^*^p ≤ 0.1, ^**^p ≤ 0.01, ^***^p ≤ 0.001.

Linear models were used to determine the changes in sorghum root and shoot dry biomass for each combination of bacterial strain, inoculation method, sampling timepoint, and degree of colonization (log copy number). Overall, all three bacteria exhibited a certain amount of root growth promotion (Figures 3A–C), with *Terrabacter* sp. being particularly stronger than the other two at enhancing root growth on week 6 after planting (Figure 3B; Table 3). The degree of growth-promotion from these bacteria was affected by the inoculation methods. Although significant root growth-promotion was measured when inoculating *Terrabacter* sp. with all five inoculation methods, the degree of growth enhancement was greater when the three seed coating methods were used (Figure 3; Table 3). On the other hand, greater root growth-promotion was detected when inoculating *C. rhizosphaerae* and *C. pinensis* with the seedling priming compared to other inoculation methods (Figure 3). In fact, root growth-promotion from *C. rhizosphaerae* was only detectable with seedling priming despite this effect being marginally significant. For *C. pinensis*, significant root growth-promotion was also observed with alginate coating and marginally significant for 12 h coating. No significant root growth-promotion was measured when inoculating *C. rhizosphaerae* and *C. pinensis* with soil drench method.

**Table 3:** Linear model testing the effect of the degree of colonization (log copy number), bacterial strain, inoculation method, and their interactions on sorghum root dry weight.

	Estimate	Std. error	*t* value	*p* value
(Intercept)	0.663	0.020	32.826	<0.001
Log copy number	−0.010	0.007	−1.425	0.155
*Chitinophaga*	−0.008	0.028	−0.282	0.778
*Terrabacter*	−0.046	0.028	−1.655	0.099
Direct coating	0.038	0.038	0.991	0.323
Alginate coating	0.071	0.030	2.378	0.018
12 h coating	0.028	0.026	1.089	0.277
Seedling priming	0.298	0.120	2.478	0.014
Soil drench	0.117	0.055	2.132	0.034
Time Week 6	0.400	0.017	23.553	<0.001
Time Week 8	0.719	0.018	41.001	<0.001
Log copy number^*^Direct coating	0.016	0.014	1.133	0.258
Log copy number^*^Alginate coating	0.006	0.012	0.537	0.592
Log copy number^*^12 h coating	0.025	0.011	2.261	0.025
Log copy number^*^Seedling priming	−0.034	0.025	−1.367	0.173
Log copy number^*^Soil drench	−0.005	0.013	−0.37	0.712
Chitinophaga^*^Direct coating	−0.047	0.034	−1.397	0.164
Terrabacter^*^Direct coating	0.069	0.035	1.994	0.047
Chitinophaga^*^Alginate coating	0.018	0.036	0.484	0.629
Terrabacter^*^Alginate coating	0.097	0.034	2.848	0.005
Chitinophaga^*^12 h coating	0.000	0.041	0.009	0.993
Terrabacter^*^12 h coating	0.105	0.035	2.995	0.003
Chitinophaga^*^Seedling priming	0.008	0.035	0.242	0.809
Terrabacter^*^Seedling priming	−0.083	0.076	−1.09	0.277
Chitinophaga^*^Soil drench	−0.019	0.034	−0.554	0.580
Terrabacter^*^Soil drench	0.029	0.042	0.698	0.486
Chitinophaga^*^Week 6	0.021	0.024	0.872	0.384
Terrabacter^*^Week 6	0.098	0.024	4.143	<0.001
Chitinophaga^*^Week 8	0.020	0.025	0.802	0.424
Terrabacter^*^Week 8	0.012	0.024	0.483	0.630

Backward selection was performed and the non-significant interactions (log copy number^*^strain, log copy number^*^sampling time, and treatment^*^sampling time) were removed from the model.

**Figure 4:**
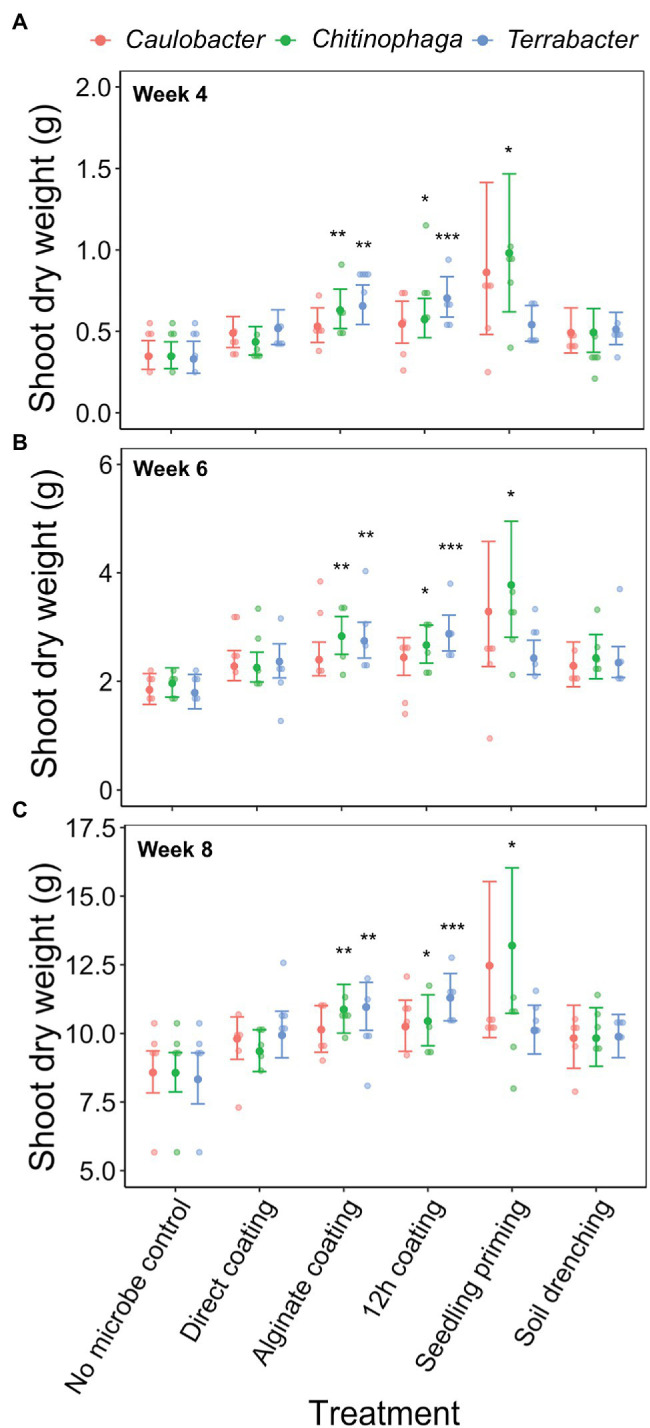
Effects of bacterial inoculation using different inoculation methods on *S. bicolor* shoot dry weight at week **(A)** 4, **(B)** 6, and **(C)** 8 after planting. Shoot dry weight was fitted to linear model and Tukey’s HSD correction was performed for multiple comparisons. Error bars and center points denote the 95% CIs and the marginal means for each strain and inoculation method combination, respectively, derived from the linear model. The distribution of raw data is represented by the dots. ^*^p ≤ 0.05, ^**^p ≤ 0.01, ^***^p ≤ 0.001.

Among the three bacteria used, only *C. pinensis* and *Terrabacter* sp. exhibited significant shoot growth enhancement (Figures 4A–C; Table 4). Significant shoot growth-promotion from *C. pinensis* was measured when inoculated with seedling priming, alginate, and 12 h coating methods. Significant shoot growth-promotion was also observed when *Terrabacter* sp. was inoculated with the same seed coating methods but not the seedling priming.

**Table 4:** Linear model testing the effect of the degree of colonization (log copy number), bacterial strain, inoculation method, and their interactions on sorghum shoot dry weight.

	Estimate	Std. error	*t* value	*p* value
(Intercept)	0.760	0.025	30.299	<2e-16
Log copy number	−0.014	0.009	−1.541	0.125
*Chitinophaga*	0.000	0.034	0.002	0.998
*Terrabacter*	−0.011	0.035	−0.308	0.758
Direct coating	0.025	0.047	0.527	0.599
Alginate coating	0.107	0.037	2.885	0.004
12 h coating	0.042	0.032	1.295	0.197
Seedling priming	0.331	0.150	2.214	0.028
Soil drench	0.073	0.068	1.063	0.289
Week 6	0.478	0.021	22.660	<2e-16
Week 8	1.192	0.022	54.715	<2e-16
Log copy number^*^Direct coating	0.022	0.017	1.282	0.201
Log copy number^*^Alginate coating	−0.003	0.015	−0.181	0.857
Log copy number^*^12 h coating	0.026	0.014	1.842	0.067
Log copy number*Seedling priming	−0.040	0.031	−1.310	0.192
Log copy number^*^Soil drench	0.003	0.017	0.203	0.840
Chitinophaga^*^Direct coating	−0.028	0.042	−0.671	0.503
Terrabacter^*^Direct coating	0.025	0.043	0.575	0.566
Chitinophaga^*^Alginate coating	0.044	0.045	0.974	0.331
Terrabacter^*^Alginate coating	0.065	0.042	1.546	0.123
Chitinophaga^*^12 h coating	0.012	0.050	0.248	0.805
Terrabacter^*^12 h coating	0.077	0.044	1.773	0.077
Chitinophaga^*^Seedling priming	0.038	0.043	0.882	0.379
Terrabacter^*^ Seedling priming	−0.115	0.094	−1.221	0.223
Chitinophaga^*^Soil drench	0.001	0.042	0.014	0.989
Terrabacter^*^Soil drench	0.020	0.052	0.388	0.699
Chitinophaga^*^Week 6	0.023	0.030	0.790	0.431
Terrabacter^*^Week 6	0.000	0.029	0.003	0.998
Chitinophaga^*^Week 8	−0.001	0.030	−0.024	0.981
Terrabacter^*^Week 8	−0.006	0.030	−0.210	0.834

Backward selection was performed and the non-significant interactions (log copy number^*^strain, log copy number^*^sampling time, and treatment^*^sampling time) were removed from the model.

### Quantification of Bacterial Colonization in Rhizosphere Under Field Condition

**Figure 5:**
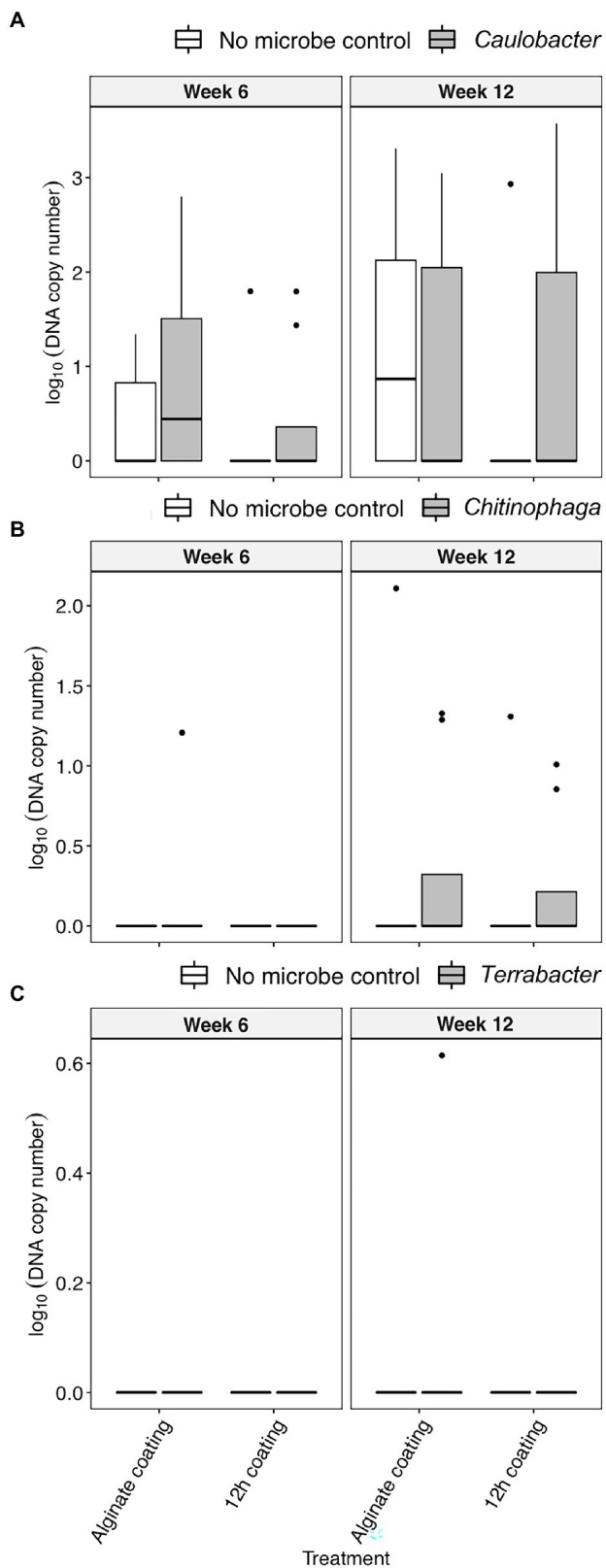
The colonization [log_10_(bacterial DNA copy number)/ng of rhizosphere DNA] of **(A)** *C. rhizosphaerae*, **(B)** *C. pinensis*, and **(C)** *Terrabacter* sp. in the rhizosphere of *S. bicolor* inoculated with alginate and 12 h coating at week 6 and 12 after planting under field condition. ANOVA was performed with Tukey’s HSD correction for multiple comparisons.

**Figure 6:**
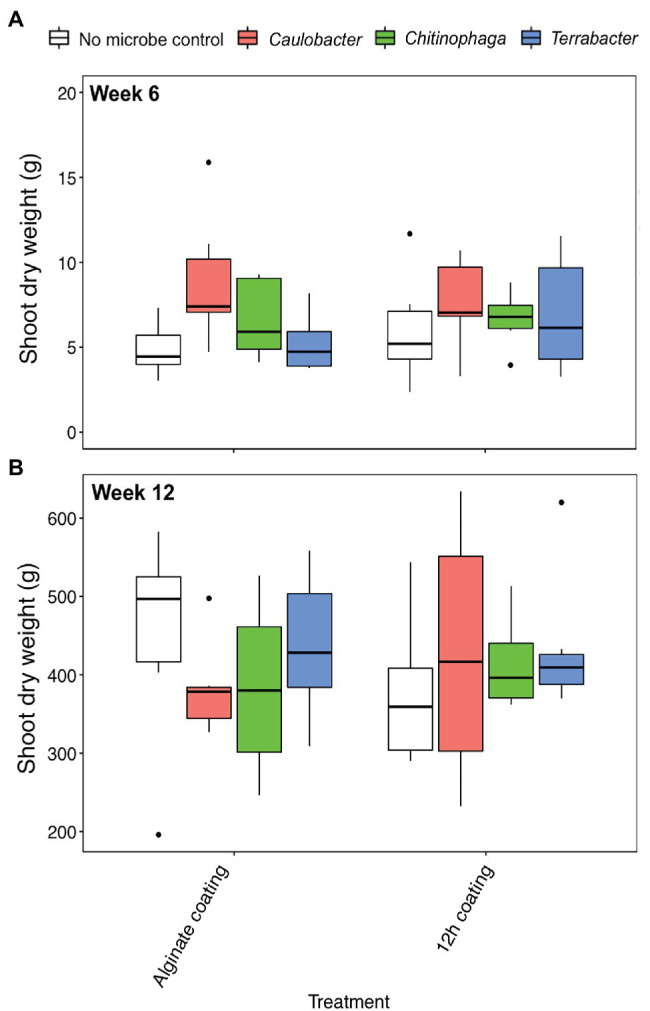
Shoot dry weight of *Sorghum bicolor* inoculated with each bacterium using alginate and 12 h coating at **(A)** week 6 and **(B)** week 12 after planting in field. ANOVA was performed with Tukey’s HSD correction for multiple comparisons. Asterisks denote significant difference in the shoot dry weight between inoculated plants and uninoculated controls.

Alginate and 12 h coating method were further tested in the field to assess their efficacy for delivering the three bacterial inoculants to sorghum rhizosphere under non-sterile conditions in which there would be competition from the native microbial communities. While *C. rhizosphaerae* and *C. pinensis* were detected in the rhizosphere up to 12 weeks after inoculation in the field, DNA copy numbers in the rhizosphere of the inoculated plants were lower as compared to that of the greenhouse experiment and not significantly different from the uninoculated control (Figures 5A,B). *Terrabacter* sp. was not detected in either of the sampling timepoints (Figure 5C). No significant improvement in shoot dry weight was measured for all bacteria and inoculation treatment combinations at both sampling timepoints (Figures 6A,B). Bacterial colonization in the root endosphere was not quantified due to the lack of biomass difference between the inoculated plants and the uninoculated controls.

## Discussion

The application of plant growth-promoting bacteria has been adopted in many countries, especially Brazil to improve crop yields and reduce the input of chemical fertilizer (Santos et al., 2019). However, there is a lack of publicly available literature that compares inoculation methods to determine which approach is more effective at delivering bacterial inoculants to the targeted plant to test their plant growth-promoting potential. To our knowledge, this is the first comprehensive study that evaluated the efficacy of multiple inoculation methods for delivering phylogenetically distinct inoculants to a cereal crop under sterile and non-sterile field conditions. We tested five inoculation methods and demonstrated that all the methods tested were successful at delivering at least one bacterial inoculant to sorghum under sterile conditions. However, the degree of plant growth-promotion from the inoculants was impacted by inoculation method. Two inoculation methods suitable for field planting, alginate and 12 h coating were tested under field condition but only a negligible amount of inoculated bacteria were detected in the rhizosphere. This may have been because the bacterial isolate concentration used to coat the seeds (10^3^–10^4^ CFU per seed) was too low to facilitate their establishment in sorghum rhizosphere under non-sterile conditions where there is competition from the natural microbial communities (Mendoza-Suárez et al., 2021). Although a standard of 10^4^ rhizobial cells per seed is widely used to inoculate legumes with medium-size seed (e.g., mung bean and pigeon pea) like sorghum (Lupwayi et al., 2000), this may not be applicable for the inoculation of non-rhizobia species and for non-legumes. On the other hand, successful colonization from inoculated bacteria was demonstrated in sorghum from a starting bacterial concentration as low as 10^2^ CFU per seed under sterile conditions (Luna et al., 2010). Despite the fact that the inoculation methods used in the field study failed to facilitate higher than background colonization levels or any growth promotion, our findings highlight the importance of testing inoculation methods under various conditions to ensure their efficacy under varying environments.

The results from the greenhouse study showed that all the bacterial inoculants used were able to persist in the sorghum rhizosphere or root until the end of the 8-week experiment. The persistence of bioinoculants over a targeted functional period is important so that their downstream impacts on plants could be sustained without needing to add another inoculant booster (Kaminsky et al., 2019). Although *C. rhizosphaerae* and *C. pinensis* were detected in the uninoculated plants on week 8, they were not observed in the controls from week 4 to week 6. We postulate that the detection of these two bacteria in the uninoculated plants may be attributed to cross-contamination between the uninoculated and inoculated samples collected at week 8. This may also be due to the amplification of closely related strains that survived the soil sterilization and colonized sorghum later in the experiment since the genera *Caulobacter* and *Chitinophaga* are ubiquitous in soil (Fulthorpe et al., 2008; Wilhelm, 2018). Although we confirmed the specificity of each primer pairs by blasting the bacterial gene fragments used to design each primer pair against the NCBI database and also ensured that they do not amplify the similar isolates in our cultural collection that were in the same genera as the targeted bacteria, there may be more closely related strains that have not been discovered and sequenced. Our findings further demonstrated that two out of the three bacteria tested were effective in promoting sorghum root and shoot growth. To gain insight into why plant biomass was enhanced, we looked more closely at the bacterial genomic sequences which suggested genes underlying plant growth-promoting functions. For example, *C. pinensis* possesses 1-aminocyclopropane-1-carboxylate deaminase gene which is important for ameliorating plant stress (Glick, 2014) while *C. pinensis* and *C. rhizosphaerae* have genes encoding siderophore synthetase and transport system that may be important in solubilizing iron in soil (Kramer et al., 2020). A follow-up *in vivo* survey will be needed to confirm the mechanisms underpinning the plant growth-promoting properties of the bacteria used in this study.

### *Caulobacter rhizosphaerae* and *Terrabacter* sp. Colonized the Rhizosphere While *Chitinophaga pinensis* Established in Both Rhizosphere and Root Endosphere

In this study, the compartmental specificity of the three inoculated bacterial isolates was demonstrated, with *C. pinensis* being the only strain that robustly colonized both the rhizosphere and root endosphere, whereas the other two were only able to colonize the rhizosphere. This result was in agreement with our expectations and other studies (Bai et al., 2015; Maggini et al., 2019) that show inoculated bacteria tend to colonize the plant compartments from which they are isolated. The degree of plant growth-promotion from *C. pinensis* was not greater than the other two bacterial isolates, although endophytic colonization could theoretically allow bacteria to interact directly with the host plant and potentially deliver the plant growth-promoting effects more efficient than the bacteria in the rhizosphere (Santoyo et al., 2016). Nonetheless, the compartmental specificity of inoculants may be crucial in determining their downstream impact on plants. For example, root nodule colonization of rhizobia is crucial for enhancing the nitrogen nutrition in the host plant because the root nodule restricts the entry of oxygen that can inhibit biological nitrogen fixation (Lindström and Mousavi, 2020).

### Generalizations About Inoculation Methods for Bacteria

Seed inoculation is currently the most widely used approach for the introduction of bioinoculants because it is the most practical and cost-effective compared to other approaches (e.g., in-furrow inoculation; O’Callaghan, 2016). Nevertheless, we found that seed coating methods may not be suitable for the inoculation of Gram-negative bacteria, which may be due to their thinner cell wall structure that renders them vulnerable to desiccation in the seed coating process (Schimel et al., 2007). In accordance with our hypothesis, the seed coating methods were less effective in delivering the Gram-negative bacteria (*C. rhizosphaerae* and *C. pinensis*) to the sorghum rhizosphere compared to seedling priming and soil drench whereas the Gram-positive strain (*Terrabacter* sp.) could be delivered successfully with seed coating. Interestingly, we also observed increased colonization from *C. pinensis* but not *C. rhizosphaerae* with the 12 h seed coating method. Since *C. pinensis* is a root endophyte, we speculate that it may have colonized the seed endophytically during the longer seed coating process (Kandel et al., 2017), thereby enhancing its survival under desiccation. Similar results were also demonstrated in another study in which 12 h seed coating promoted the initial rhizosphere colonization of an endophytic biocontrol bacterium, *Serratia plymuthica* HRO-C48, on oilseed rape and enhanced the survival of this bacterium on seeds (Müller and Berg, 2007). Different inoculation methods may further affect the downstream impacts of inoculants on plants. For instance, seed inoculation of Gram-positive *Bacillus* strains on cowpea and mash bean has been shown to suppress root-infecting phytopathogens more effectively than soil drench (Dawar et al., 2010). The improved biocontrol abilities from these inoculants with seed inoculation may be attributed to the fact that seed inoculation allowed them to establish inside the root prior to pathogen infestation. On the other hand, soil drench has been shown to be more effective in delivering inoculants to Italian ryegrass growing on soil contaminated with diesel oil than the 12 h seed coating method, and improved plant growth-promotion and hydrocarbon degradation (Afzal et al., 2012). This was probably due to the greater density of inoculant being applied from the soil drench than the 12 h coating method in which the amount of bacteria applied to seeds was constrained by the seed size. These findings suggest that it may be important to tailor inoculation methods for inoculants with specific characteristics and functionalities.

### Colonization Rates Are Not Linked to Growth Promotion

Although all the inoculation methods tested successfully delivered bacterial isolates to the sorghum rhizosphere in the greenhouse study, the impacts on sorghum growth were variable. Despite being equally effective at delivering *C. pinensis* to sorghum rhizosphere, no growth-promotion was observed with the soil drench method while both shoot and root growth promotions were detected with seedling priming. Similarly, *Burkholderia ambifaria* MCI 7 was reported to improve maize growth when coated on seed but was detrimental to growth when applied into the soil (Ciccillo et al., 2002). Although it was unclear why the same bacterium had contrasting effects on plant growth, the authors noted that the root adjacent to the stem was mainly colonized when the seed was coated whereas the entire root system was colonized with the soil drench method (Ciccillo et al., 2002). In our studies, *Terrabacter* sp. was in lower abundance in the rhizosphere but was still able to promote sorghum root growth comparable to or better than the other two bacteria. This is in line with another study that observed similar biomass-promoting effects on banana from *Pseudomonas fluorescens* Ps006 and *Bacillus amyloliquefaciens* Bs006 despite *P. fluorescens* Ps006 being a less efficient root colonizer compared to *B. amyloliquefaciens* Bs006 (Gamez et al., 2019). These findings highlight that the degree of colonization by inoculated bacteria of host plants may not necessarily be directly related to the level of growth enhancement induced.

## Conclusion

This study compared several different bacterial inoculation methods to determine the most suitable approach for studies of the impact of bacterial inoculation of plants grown in sterilized greenhouse soil experiments and in the field. Simply coating seeds with a bacterial suspension was suitable for the inoculation and successful colonization of Gram-positive bacteria in the greenhouse, whereas the field results were inconclusive. For Gram-negative bacteria direct inoculation using seedling priming or soil drench led to higher colonization efficiency than seed coating. The method of inoculation was critical in these types of experiments because the colonization rates and plant growth-promoting potential of inoculants were influenced by inoculation method. These findings show that the inoculation methods should be tailored to accommodate the characteristics of different bacterial inoculants to ensure successful colonization of the targeted plant species.

## Data Availability Statement

The datasets presented in this study can be found in online repositories. The names of the repository/repositories and accession number(s) can be found in the article/supplementary material.

## Author Contributions

DS contributed in conceptualization of experiment and revisions of the manuscript. YNC performed the experiment and statistical analyses and wrote the manuscript. SF helped with planting and maintaining the field, as well as sampling. All authors contributed to the article and approved the submitted version.

## Funding

This research was funded by United States Department of Energy BER, Grant/Award Number: DE-SC0014395 and the University of Nebraska - Lincoln Agricultural Research Division.

## Conflict of Interest

The authors declare that the research was conducted in the absence of any commercial or financial relationships that could be construed as a potential conflict of interest.

## Publisher’s Note

All claims expressed in this article are solely those of the authors and do not necessarily represent those of their affiliated organizations, or those of the publisher, the editors and the reviewers. Any product that may be evaluated in this article, or claim that may be made by its manufacturer, is not guaranteed or endorsed by the publisher.
