# Supplementary material for: Assessment of Bacterial Inoculant Delivery Methods for Cereal Crops
Source: Front Microbiol. 2022 Jan 26;13:791110. doi: 10.3389/fmicb.2022.791110 (PMC8826558; doi:10.3389/fmicb.2022.791110)
Supplement: Supplementary file 1 [file Table_1.docx]

All microbes that we used in the manuscript have been genome-sequenced and their 16S rRNA data can be found on the repository of U.S. Department of Energy Joint Genome Institute under the corresponding taxon ID:

| **Bacteria strain** | **Joint Genome Institute taxon ID** |
| --- | --- |
| *Chitinophaga pinensis* | 2818991442 |
| *Caulobacter rhizosphaerae* | 2818991454 |
| *Terrabacter* sp. | 2818991462 |
| *Caulobacter segnis*1776 | 2928531327 |
| *Caulobacter rhizosphaerae*2154 | 2918824543 |
| *Chitinophaga pinensis*1232 | 2821136567 |
| *Chitinophaga sancti*3198 | 2904467357 |
| *Chitinophaga pinensis*1209 | 2818991460 |
| *Terrabacter*sp. 3211 | 2818991458 |
| *Terrabacter lapilli* 3265 | 2818991469 |
